# What makes a multidisciplinary medication review and deprescribing intervention for older people work well in primary care? A realist review and synthesis

**DOI:** 10.1186/s12877-023-04256-8

**Published:** 2023-09-25

**Authors:** Eloise Radcliffe, Renée Servin, Natalie Cox, Stephen Lim, Qian Yue Tan, Clare Howard, Claire Sheikh, Paul Rutter, Sue Latter, Mark Lown, Lawrence Brad, Simon D. S. Fraser, Katherine Bradbury, Helen C. Roberts, Alejandra Recio Saucedo, Kinda Ibrahim

**Affiliations:** 1 School of Primary Care, Population Sciences and Medical Education, Faculty of Medicine, University of Southampton, Aldermoor Health Centre, Aldermoor Close, Southampton, SO16 5ST UK; 2 NIHR Applied Research Collaboration ARC Wessex, University of Southampton, Southampton, UK; 3 Faculty of Medicine, Imperial College London, South Kensington Campus, London, UK; 4 Academic Geriatric Medicine, Faculty of Medicine, University of Southampton, Southampton, UK; 5 Wessex Academic Health Science Network, Science Park, Chilworth, Southampton, UK; 6 Hampshire and Isle of Wight Integrated Care Board, Southampton, UK; 7 School of Pharmacy and Biomedical Sciences, Portsmouth University, Portsmouth, UK; 8 School of Health Sciences, University of Southampton, Southampton, UK; 9 Westbourne Medical Centre, Westbourne, Bournemouth, UK; 10 NIHR Southampton Biomedical Research Centre, University Hospital Southampton Foundation Trust, Southampton, UK; 11 School of Psychology, University of Southampton, Southampton, UK; 12 School of Healthcare Enterprise and Innovation, Trials and Studies Coordinating Centre, National Institute of Health Research Evaluation, University of Southampton, Southampton, UK

**Keywords:** Medicines optimisation, Deprescribing, Polypharmacy, Older people, Primary care, Multidisciplinary team, Realist review

## Abstract

**Background:**

A third of older people take five or more regular medications (polypharmacy). Conducting medication reviews in primary care is key to identify and reduce/ stop inappropriate medications (deprescribing). Recent recommendations for effective deprescribing include shared-decision making and a multidisciplinary approach. Our aim was to understand when, why, and how interventions for medication review and deprescribing in primary care involving multidisciplinary teams (MDTs) work (or do not work) for older people.

**Methods:**

A realist synthesis following the Realist And Meta-narrative Evidence Syntheses: Evolving Standards guidelines was completed. A scoping literature review informed the generation of an initial programme theory. Systematic searches of different databases were conducted, and documents screened for eligibility, with data extracted based on a Context, Mechanisms, Outcome (CMO) configuration to develop further our programme theory. Documents were appraised based on assessments of relevance and rigour. A Stakeholder consultation with 26 primary care health care professionals (HCPs), 10 patients and three informal carers was conducted to test and refine the programme theory. Data synthesis was underpinned by Normalisation Process Theory to identify key mechanisms to enhance the implementation of MDT medication review and deprescribing in primary care.

**Findings:**

A total of 2821 abstracts and 175 full-text documents were assessed for eligibility, with 28 included. Analysis of documents alongside stakeholder consultation outlined 33 CMO configurations categorised under four themes: 1) HCPs roles, responsibilities and relationships; 2) HCPs training and education; 3) the format and process of the medication review 4) involvement and education of patients and informal carers. A number of key mechanisms were identified including clearly defined roles and good communication between MDT members, integration of pharmacists in the team, simulation-based training or team building training, targeting high-risk patients, using deprescribing tools and drawing on expertise of other HCPs (e.g., nurses and frailty practitioners), involving patents and carers in the process, starting with ‘quick wins’, offering deprescribing as ‘drug holidays’, and ensuring appropriate and tailored follow-up plans that allow continuity of care and management.

**Conclusion:**

We identified key mechanisms that could inform the design of future interventions and services that successfully embed deprescribing in primary care.

**Supplementary Information:**

The online version contains supplementary material available at 10.1186/s12877-023-04256-8.

## Background

A third of older people aged 65 and over take five or more regular medications, widely referred to as polypharmacy [1]. Polypharmacy can cause a significant but avoidable burden and source of harm for patients and places strain on healthcare systems [2]. Polypharmacy in older people is associated with increased potentially inappropriate medications (PIMs), which refers to whether a drug is safe or unsafe in terms of its pharmaceutical properties but also encompasses the assessment of older persons’ prescription medications in the context of their multiple co-morbidities, complex medication regimes, functional and cognitive status, treatment goals and life expectancy [3]. PIMs can increase risk of falls, cognitive impairment, functional decline, hospital admission and death [4–6] and these effects can be amplified in those living with frailty [7].

Management of polypharmacy involves medicines optimisation commonly defined as ‘a person-centred approach to safe and effective medicines use, to ensure people obtain the best possible outcomes from their medicines’ [8]. A core part of medicines optimisation is deprescribing which involves tapering /dose reduction, stopping, or switching drugs with the goal of improving outcomes [9]. Research has shown that deprescribing is feasible and safe across a wide range of conditions, medications, settings and with the use of different deprescribing tools [10–16], and can lead to a reduction in polypharmacy and PIMs [17, 18].

A primary care setting is ideal for conducting regular structured medication reviews, as it is the first point of contact with health services and gatekeeper to other specialist services for most patients in many European countries [19]. General Practitioners (GPs) are largely responsible for the management of patients with long-term health conditions and have access to patients’ medical records to support any decisions related to treatments. Yet medication reviews and deprescribing do not happen routinely due to GPs’ lack of time, increased workloads and worries about stopping medications, especially if prescribed by other physicians [20]. Involving other non-medical prescribers such as pharmacists and advanced nurse practitioners in reviewing medications has been suggested to address the barriers to deprescribing [21]. Pharmacists have the knowledge and skills in managing medications and they are ideally placed to lead medication reviews [22]. Literature suggests that structured medication reviews to identify and reduce or stop inappropriate medications should be underpinned by a multidisciplinary approach and shared-decision making between health care professionals (HCPs) and patients [8, 20, 23–26]. A recent realist review of person-centred medication review and deprescribing in older people also identified continuity of care and the development of trust as essential to successfully deprescribe inappropriate medications [27].

The current realist review focuses specifically on multidisciplinary deprescribing in the context of primary care. The benefits of a multidisciplinary approach in general within primary care are well recognised including improved patient care through increased opportunities for sharing knowledge and ideas, and a sense of partnership, with many factors influencing the effectiveness of teamwork including dedicated time and resources, co-location and staff commitment [28]. In the UK, pharmacists and other HCPs including nurses and physiotherapists, are increasingly working as independent non-medical prescribers within a multidisciplinary primary care team to consult with and treat patients directly and more countries are moving towards this model [29]. Evidence indicates that multidisciplinary interventions and those involving pharmacists are effective in reducing inappropriate prescribing, but further research is essential to explore how this sharing of responsibilities could work in practice [2, 25, 26]. Deprescribing requires complex changes to established patterns of behaviour at the individual, organisational, and systems levels. Researchers have identified the need to understand how deprescribing works, for whom and how to sustain its implementation in clinical practice [30]. Our aim was to develop a programme theory to inform recommendations for successful implementation of multidisciplinary deprescribing for older people within the context of primary care.

## Methods

Realist approaches are interested in the behaviours of those involved in an intervention, the influential factors that bring about or prevent those behaviours; and the intended and unintended outcomes that result from implementing an intervention [31]. Realist methods explore the causal links between the context in which an intervention takes place, the mechanisms or responses which are triggered by the intervention in specific contexts and certain outcomes. This is a theory-driven method which follows a non-linear, iterative process of analysis and interpretation, and focuses on understanding how mechanisms are shaped and constrained by social context. Researchers seek to establish *what works, for whom, under what circumstances* and *why* [32]*.* Realist methods are concerned with building hypothetical explanations or ‘programme theories’ that aim to capture the mechanisms underlying complex interventions in the contexts in which they work [33].

This realist review is part of a larger programme of work that aims to develop and test a complex multidisciplinary deprescribing intervention in primary care targeting older people (the MODIFY study). When developing and piloting an intervention, theorising the contextual conditions necessary for intervention mechanisms to work is essential [34]. Where interventions are scaled up and translated into routine practice, a realist approach is valuable in uncovering an understanding of longer-term sustainability, benefits and challenges [34]. The iterative nature of the realist methodologies means moving backwards and forwards dynamically between reviewing evidence, drawing on existing literature and working with stakeholders.

Our realist review followed the key steps outlined by Pawson et al. [31], clarifying the scope, searching for the evidence and extracting data, and synthesising the evidence and drawing conclusions. The current consensus methodological standards for realist syntheses developed by the Realist And Meta-narrative Evidence Synthesis: Evolving Standards (www.rameses project.org) [35] was also used as a basis for conducting this review. Our findings are reported using the RAMESES guidelines checklist (see supplementary file 2).

### Step 1: Clarifying the scope

Realist reviews begin with the development of initial programme theories that explain how an intervention works based on Context, Mechanism and Outcomes Configurations (CMOCs): an intervention that in certain contexts (C), due to the operation of some underlying causal forces or mechanism (M), leads to a particular outcome (O). More specifically, mechanisms refers to the resources offered through an intervention and the way people respond to those resources (eg. information, engagement, training) [32]. We developed ten initial CMOCs through a scoping review of the literature and iterative discussion and consultation with the study Research Management Group (RMG) (see supplementary file 3). The RMG group included key stakeholders; clinical and academic pharmacists, general practitioners, nurses, geriatricians and methodologists (three members had training and experience in realist synthesis). The scoping search of the literature was based on the RMG members’ prior knowledge of key published papers on deprescribing interventions and a PubMed search. At this stage, the literature was not analysed in-depth but used to provide an overview to inform the generation of initial CMOCs. The group met twice to discuss and revise the initial programme theories based on their clinical and academic expertise and experience. Our initial programme theory highlighted the main mechanisms that could facilitate or inhibit MDT processes for deprescribing among older people in primary care.

### Step 2: Searching for the evidence

**Fig. 1:**
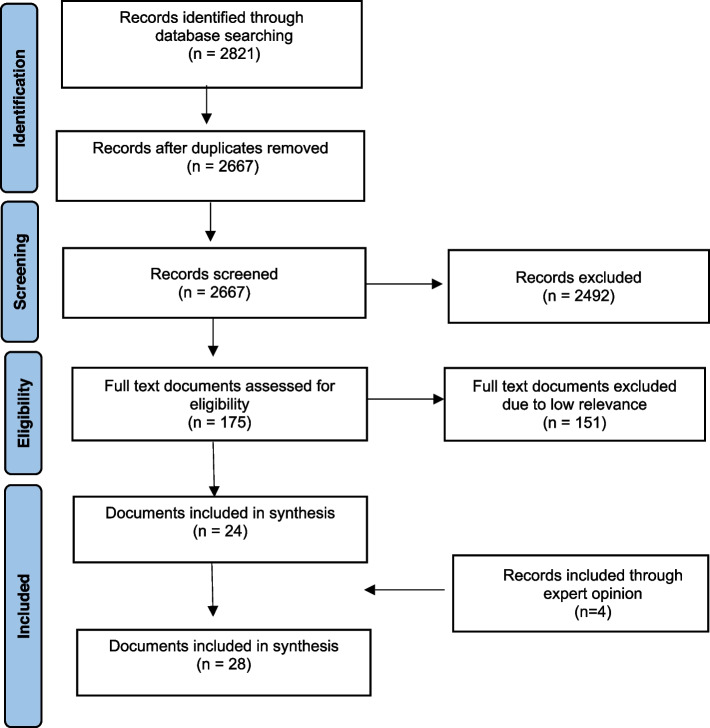
PRISMA Document selection flowchart [36]

Based on the initial programme theory, we developed our search strategy to identify relevant literature on interventions that employ a multidisciplinary approach to medication review with a deprescribing element, aimed at people aged 65 and over (and their carers) within a primary care setting. A librarian-guided literature search was conducted using a search strategy (see supplementary file 1). In March 2022 we searched the following databases: Medline, EMBASE, CINAHL, PubMed, Web of Science, PsycINFO and Cochrane Library, NICE guidelines and grey literature searches via google, google scholar and social media (e.g. Twitter). We also examined the reference lists of included relevant documents to identify additional documents. In addition, we continued to include new papers identified through social and professional networks and journals alerts (See Fig. 1).

### Step 3: Selecting articles and extracting data

Inclusion and exclusion criteria for the review (See Table 1) were based on our research question, initial CMOCs and discussion with the RMG. Those aged 65 and over were included, in line with the majority of other research studies focusing on older people. Care home residents were excluded as their medication is frequently managed with the assistance of care home staff, in comparison to people living in their own home who often manage medication on their own or with the help of a family member or friend (informal carer). We included documents if they described deprescribing interventions or medication review interventions with a focus on deprescribing. We only included interventions that employed an MDT approach- defined as a group of HCPs from two or more disciplines brought together to determine patients’ treatment plan [23], which can be either independently or in parallel [22].

**Table 1:** Inclusion and exclusion criteria for the realist review

	**Inclusion criteria**	**Exclusion criteria**
Context	Interventions conducted in general practice alone or in combination with community pharmacy or home visits by any HCP	Any other setting eg. secondary care or care homes
Population	Participants aged 65 years and over	Participants aged under 65 years
	Participants living in their own home	Participants living in nursing/care homes
Intervention	Intervention for medication review with a deprescribing element (i.e., the process of tapering /dose reduction, stopping, or switching drugs, with the goal of improving outcomes)	Intervention for medication review without a clear deprescribing element
	Intervention includes a multidisciplinary aspect (i.e., a group of professionals from two or more disciplines who work on the same project or issue, independently or in parallel)	Intervention includes professionals from a single discipline (i.e., without a multidisciplinary aspect)
Outcome	Any process or staff/patient outcome measures based on primary data related to deprescribing	No process or outcome measure reported
Other	Studies published in English	Studies not published in English

All of the identified abstracts were double-screened for eligibility by one author (ER) who screened all abstracts, and one of four other authors (KI, QYT, SL, NC). The blinded RAYYAN software was used for this task and also for deduplication. Any discrepancies were resolved through discussion between the two authors. The next stage involved four of the authors (KI, ER, ARS, RS) double-screening all full-text documents that were identified as potentially eligible at this stage, with any discrepancies regarding inclusion resolved through regular team discussion between all four authors. The selection of full-text documents primarily focused on the extent to which the articles could contribute to the development and refinement of the initial programme theory. When the final list of full text documents to be included had been agreed on by the research team, the characteristics and details of these interventions and populations were extracted into an MS Excel spreadsheet. An overview of all studies included is shown in Table 2 and the document selection process is shown in Fig. 1 in the PRISMA flowchart [36].

**Table 2:** Documents included in the realist review (n = 28)

Author Year Country	Study design/ methods	Intervention	Sample and participants	Objectives
Bryant, L. J., et al., (2010) [37] New Zealand	Randomised Controlled Trial (RCT)	Community pharmacists undertook a clinical medication review (Comprehensive Pharmaceutical Care) and met with the patient’s general practitioner (GP) to discuss recommendations about possible medicine changes	198 patients, aged 65 and over, taking 5 medicines or more	To determine whether involvement of community pharmacists undertaking clinical medication reviews, working with general practitioners, improved medicine-related therapeutic outcomes for patients
Campins, L., et al. (2017) [38] Spain	RCT	The intervention consisted of 3 consecutive phases 1. A trained, experienced clinical pharmacist evaluated all drugs prescribed to each patient using the GP–GP algorithm and appropriateness based on the STOPP/START criteria 2. The pharmacist discussed recommendations for each drug with the patient’s GP to come up with a final set of recommendations 3. Recommendations were discussed with the patient, and a final decision was agreed by GP and their patients in a face-to-face visit	503 patients, aged 70 and over, taking 8 medicines or more	To assess the effectiveness and safety of a medication evaluation programme for community-dwelling polymedicated elderly people
Cardwell, K., et al. (2020) [39] Ireland	Non-randomised pilot study	A pharmacist joined the practice team for 6 months (10 h/week) and undertook medication reviews (face to face or chart based) for adult patients, provided prescribing advice, supported clinical audits and facilitated practice-based education	786 patients, aged 65 years and over, taking 10 repeat medicines or more	To develop and assess the feasibility of an intervention involving pharmacists, working within general practices, to optimise prescribing in Ireland
Clark, C. M., et al. (2003) [40] USA	Evaluation of a pilot program	Community pharmacy-based clinical pharmacist provided face-to-face medication reviews for patients over 65 years old as part of their Annual Wellness Visit with a focus on deprescribing PIMs. No clinical pharmacy service existed at the practice when this program was implemented	21 patients aged 65 years and over, living in the community	To develop and pilot-test a model in which a community-based clinical pharmacist was incorporated as part of a Medicare Annual Wellness Visit to make deprescribing recommendations targeted at PIMs in seniors
Mecca, M. C., et al. (2019) [41] USA	A prospective cohort study with an internal comparison group	The Initiative to Minimize Pharmaceutical Risk in Older Veterans (IMPROVE) polypharmacy clinic was created as an interprofessional education intervention to provide a platform for teaching internal medicine and nurse practitioner residents about outpatient medication management and deprescribing for older adults	36 patients, aged 65 years and over, taking 10 medicines or more	To assess patients’ knowledge of polypharmacy and perceptions of an interprofessional education intervention
Lenaghan, E., et al. (2007) [42] UK	RCT	The intervention comprised two home visits by a community pharmacist who educated the patient/carer about their medicines, noted any pharmaceutical care issues, assessed need for an adherence aid, and subsequently met with the lead GP to agree on actions	41 patients, aged 80 years and over, living at home, taking four or more medicines, with at least one additional medicines-related risk factor	To assess whether home-based medication review by a pharmacist for at-risk older patients in a primary care setting can reduce hospital admissions
Leendertse, A. J., et al. (2013) [43] The Netherlands	An open controlled multicentre study	The intervention consisted of a patient interview, a review of the pharmacotherapy and the execution and follow-up evaluation of a pharmaceutical care plan. The patient’s own pharmacist and GP carried out the intervention	674 patients with a high risk of medication-related hospitalizations based on age 65 years and over), use of five or more medicines, non-adherence and type of medication used	To investigate the effect of a multicomponent pharmaceutical care intervention on these outcomes
Lenander, C., et al. (2014) [44] Sweden	RCT	Patients answered a questionnaire regarding medications. The pharmacist reviewed all medications (prescription, non-prescription, and herbal) regarding recommendations and renal impairment, giving advice to patients and GPs. Each patient met the pharmacist before seeing their GP	209 patients aged 65 years and over with five or more different medications	To determine whether a pharmacist-led medications review in primary care reduces the number of drugs and the number of drug-related problems
Khera, S., et al. (2019) [45] Canada	Quasi-experimental pre-test-post-test design	A structured pharmacist-led medication review using evidence-based explicit criteria (ie, Beers and STOPP/START criteria) and implicit criteria (i.e., pharmacist expertise) for potentially inappropriate prescribing	54 patients aged 65 years and over with frailty who have polypharmacy and/or 2 or more chronic conditions (ie, high-risk group for drug-related issues)	To assesses the impact of a team-based, pharmacist-led structured medication review process in primary care on the appropriateness of medications taken by older adults living with frailty
Hazen, A. C. M., et al. (2019) [46] The Netherlands	An observational cross-sectional study	Clinical medication reviews led by non-dispensing pharmacists. The medication review started with a semi-structured interview with the patient identifying the patient’s experiences, needs and concerns about medication. These were integrated with the medical records to determine potential drug therapy problems. The pharmacist developed a pharmaceutical care plan in collaboration with the patient and the GP, including recommendations to stop, start or switch medication, to adjust dosages or to improve adherence to medication. The recommendations were implemented and monitored, mainly by the pharmacist	270 patients aged 65 years and over using 5 or more chronic medications	To evaluate the process of clinical medication review for elderly patients with polypharmacy performed by non-dispensing pharmacists embedded in general practice. The aim was to identify the number and type of drug therapy problems and to assess how and to what extent drug therapy problems were actually solved
Foubert, K., et al. (2019) [47] Belgium	Prospective observational study	Participating pharmacists received protocol training. Pharmacists conducted a face-to-face intermediate medication review based on medication history and patient information, according to Pharmaceutical Care Network Europe typology of medication reviews using the Ghent Older People's Prescriptions community Pharmacy Screening (GheOP3S)‐tool and other sources of information if necessary	75 patients aged 70 and over, using five or more medications	To describe the characteristics of the detected drug related problems (DRPs) and the subsequent pharmacists' recommendations with their acceptance and implementation rate resulting from a pharmacist‐led medication review using the Ghent Older People's Prescriptions community Pharmacy Screening (GheOP3S)‐tool (an explicit screening tool to detect DRPs) and to assess the potential impact of the intervention
Fixen, D. R., et al. (2022) [48] USA	Retrospective review	Patients were mailed an education packet of information that included working with the clinical pharmacy team, behavioural health team, or both in order to deprescribe their sedative-hypnotic medication	93 ‘Older adults’ with a prescription filled in the prior 12 months for a sedative-hypnotic medication, benzodiazepine or nonbenzodiazepine sedative-hypnotic (ie, zolpidem, zaleplon, eszopiclone) were included	To evaluate the outcome of a multidisciplinary intervention tailored to older adult patients and their primary care providers (PCP)
Verdoorn, S., et al. (2019) [49] The Netherlands	RCT	A clinical medication review (CMR) focused on personal goals using Goal Attainment Scaling (GAS), starting with a face-to-face patient interview by the pharmacist	315 patients aged 70 years or more using 7 or more medications	The aim of this study was to investigate whether GAS is a useful tool for determining goals and monitoring their attainment during CMR
Williams, M. E., et al. (2004) [50] USA	RCT	A comprehensive review by a consultant pharmacist and recommended modification of a patient’s medication regimen. Changes were endorsed by each patient’s primary physician and discussed with each patient	133 patients aged 65 and over taking five or more medications	To determine whether a medication review by a specialized team would promote regimen changes in elders taking multiple medications and to measure the effect of regimen changes on monthly cost and functioning
Fiss T., et al. (2013) [51] Germany	We conducted a prospective non-randomized implementation cohort study	The intervention was implemented by a three -party healthcare team (practice assistant, pharmacist, GP)) and adherence supporting strategies (using a medication reminder chart, medication compliance aid). It comprised pharmaceutical care (and follow-up visit) by the local pharmacy in addition to medical interventions by the GP	408 patients, aged 65 and over with ‘any intake of drugs'	To answer the following questions: -Is home medication review in a tripartite setting, with subsequent pharmaceutical and medical interventions, effective in reducing specific DRPs in a German rural area, and how many drugs are the patients taking? -Is it possible to improve adherence supporting strategies with a medication plan / medication reminder chart and a medication box?
Stuhec, M. (2021) [52] Slovenia	A pilot trial	The model for Slovenian primary care settings had teams of one clinical pharmacist and all GPs from a primary care setting (e.g., 10 GPs and one pharmacist). Teams consisted of all GPs at a primary care setting and a clinical pharmacist working in the GPs’ offices. GPs could refer patients to the clinical pharmacist for a medication review. The clinical pharmacist prepared a medication review and final recommendations which were sent back to the GPs. The GPs could accept or reject the recommendations at the patient’s next regular visit	48 patients, aged 65 and over	To evaluate a programme in Slovenia to reduce medication-related problems and polypharmacy in the older adult with polypharmacy
Van der Meer, H. G., et al. (2018) [53] The Netherlands	RCT	A medication review by the community pharmacist in collaboration with the patient’s GP and patient	305 patients aged 65 years and over who used 5 or more medicines for 3 months or more, including at least one psycholeptic/psychoanaleptic medication and who had a Drug Burden Index (DBI) of 1 or more	To evaluate if a pharmacist-led medication review is effective at reducing the anticholinergic/ sedative load, as measured by the DBI
Van der Meer, H. G., et al. (2019) [54] The Netherlands	Prospective study	Information technology (IT) based tool developed by researchers aimed at identifying patients at risk of newly prescribed anticholinergic drug use, these patients would then be reviewed by a community pharmacist whose recommendations were brought to the GP. This was then presented to the patient by either pharmacist or GP	157 patients, aged 65 years and over, with existing high anticholinergic/sedative loads (drug burden index 2 or more) and a newly initiated anticholinergic/sedative medication	To explore the feasibility, acceptability and potential effectiveness of an innovative IT-based intervention to prevent an increase in anticholinergic/sedative load in older people
Oboh L., et al. (2018) [55] UK	Evaluation based on systematic anonymised data recorded regarding full operation of the integrated care clinical pharmacist service in its first 15 months	Community Matrons (CMs) identified patients who were experiencing medicines related problems. These were referred to the integrated care clinical pharmacist who undertook a full medication review at the patient’s home and recorded activities, which were independently analysed anonymously	143 patients. ‘Frail, older patients with complex medicines-related needs living in their own homes’. CMs identified and referred those patients from their active caseload who were experiencing actual medicines-related problems or those who had other challenges affecting medicine-taking. There were no exclusion criteria	To describe a new specialist pharmacy service (called the integrated care clinical pharmacist) in terms of how it works, what it achieves and its policy implications
Parkinson, L., et al. (2021) [56] Australia	Pilot study	A pharmacist home review and discussion of patients’ medications, communication between pharmacist and GP, and a GP–patient discussion, all facilitated through AusTAPER. AusTAPER integrates patient priorities, decision- support tools to electronically flag potentially inappropriate medicines and a clinical pathway for structured assessment and follow-up by both community pharmacist and GP in a web-based system	Nine patients and two GPs responded. (Patients aged 70 years or older, taking at least five regular medicines)	The objective of this study was to explore the Australian general practitioner (GP) and patient experience of AusTAPER, a pharmacist facilitated web-based deprescribing tool, within a pilot implementation of the tool
Romskaug, R., et al. (2019) [57] Norway	Cluster randomized, single-blind, clinical trial	The intervention consisted of 3 main parts: (1) clinical geriatric assessment of the patients combined with a thorough review of their medications; (2) a meeting between the geriatrician and the family physician (FP); and (3) clinical follow-up	174 patients, home-dwelling patients 70 years or older, using at least 7 medications regularly, and having their medications administered by the home nursing service	To investigate the effect of clinical geriatric assessments and collaborative medication reviews by geriatrician and FP on health-related quality of life and other patient-relevant outcomes in home-dwelling older patients receiving polypharmacy
Dalin, D. A., et al. (2019) [58] Denmark	Evaluation	Medication review in general practice by an interdisciplinary medication team of pharmacists and physicians, based on information concerning medication, diagnosis, relevant laboratory data and medical history supplied by the general practitioner. The medication review was discussed with the patients’ general practitioners and feedback was received from them regarding acceptance rates of the recommended changes	94 patients, aged 65 years and over, using 6 or more medications	The study objective is to describe and evaluate a method for conducting medication review in general practice by an interdisciplinary medication team of pharmacists and physicians
Denneboom, W., et al. (2007) (a) [59] The Netherlands	RCT	Pharmacists were randomised and the pharmacists in both intervention groups performed treatment reviews with the support of the computerised screening tool. They had to decide which of the recommendations highlighted by the tool should be given to the GP, and whether additional recommendations concerning the pharmacotherapy of these patients should be highlighted. The two intervention groups differed in their organisational models (written-feedback by pharmacists to GP vs case-conference between the pharmacist and GP)	738 patients, aged 75 years and over taking five or more medicines	To determine which procedure for treatment reviews (case conferences versus written feedback) results in more medication changes, measured at different moments in time. To determine the costs and savings related to such an intervention
Denneboom, W., et al. (2007) (b) [60] The Netherlands	Written questionnaires, structured telephone interviews	As above (Process evaluation for the study above)	64 GPs and 28 pharmacists responded to a questionnaire. 18 Pharmacists and 16 GPs participated in telephone interviews	The aim of the study was to describe the feasibility of two methods for treatment review (results were given to the GP either in case conferences or in written feedback), and to determine if and how the process of treatment review can be improved
Bayliss E. A., et al. (2022) [61] USA	Cluster RCT	An educational brochure and a questionnaire on attitudes toward deprescribing were mailed to patients prior to a primary care visit, clinicians were notified about the mailing, and deprescribing tip sheets were distributed to clinicians at monthly clinic meetings	3012 patients aged 65 years or older with dementia or mild cognitive impairment who had 1 or more additional chronic medical conditions and were taking 5 or more long-term medications	To examine the effectiveness of increasing patient and clinician awareness about the potential to deprescribe unnecessary or risky medications among patients with dementia or mild cognitive impairment
Sheehan et al. O.C., (2022) [62] USA	Qualitative interviews and surveys with stakeholders	As above (Process evaluation for the study above)	15 patients, 7 caregivers, and 28 clinicians, participating in the above study	To examine the effectiveness of increasing patient and clinician awareness about the potential to deprescribe unnecessary or risky medications among patients with dementia or mild cognitive impairment
Trenaman S. C., et al. (2022) [63] Canada	The evaluation included measures of medication appropriateness, patient satisfaction, and healthcare professional satisfaction	Pharmacist-led deprescribing in collaborative primary care settings using the seven components of knowledge translation. Patient and stakeholder engagement shaped the deprescribing intervention. The seven essential components of knowledge translation include identification of a problem, adaptation of knowledge to local context, assessment of barriers to knowledge use, selection, tailoring, and implementation of an intervention, monitoring knowledge use, evaluation of outcomes, and sustaining knowledge use	13 patients, across the three sites. Patients who have stable management of all chronic conditions, taking medications from a targeted drug list and have not had a change in the targeted medication for the past three months	To describe implementation of pharmacist-led deprescribing in collaborative primary care settings using the seven components of knowledge translation
Jamieson H., et al. (2023) [64] New Zealand	RCT	Pharmacists provided deprescribing recommendations by letter to GPs. Each participant experienced a home-based pharmacist-conducted medication review. All pharmacists received training specific to the intervention and followed the deprescribing guidelines defined in the study protocol. The pharmacist did not have access to the participants’ clinical notes or medication records and all clinical decision-making, including prescribing, remained with the GP	363 patients, aged 65 and over, identified as frail based on a needs assessment, taking at least 1 medication with anticholinergic or sedative effects regularly at the minimum registered daily adult dose (which would result in DBI of 0.5 or above)	To test, by conducting an RCT, whether patient-specific deprescribing recommendations developed by pharmacists following a medication review and provided to the patient’s GP reduced the use of anticholinergic and sedative medications. In addition, we hypothesized that any resulting reduction in DBI score would be more pronounced for older adults with a greater level of frailty

All documents were assessed based on relevance and rigour, in line with RAMESES guidelines [35]. Relevance was determined based on the extent to which the data can contribute to the development of the programme theories i.e. there was sufficient description of the intervention to determine contextual information and mechanisms. We only included papers that provided this detail. Rigour refers to the quality and credibility of the methods used to generate the data. All the documents included focused on the testing or evaluation of interventions and the methodological rigour in terms of how the data were generated was high for all the studies. We also made the decision that if the intervention did not report outcomes, it was excluded as it would not address our specific aims (i.e. not relevant). Therefore, the majority of documents included were of high relevance and high rigour. For three documents which were assessed as highly relevant but lacked adequate detail, we contacted the authors for clarification and collected additional detail where possible. The relevance and rigour of all papers included were assessed by ER, KI, AR and RS and any discrepancies were resolved in discussion as a team.

### Step 4: Synthesising the evidence and drawing conclusions

Extracted data was coded by four authors (ER, KI, AR, RS) using detailed tables in Excel to identify context, mechanisms and outcomes in each intervention, taking an inductive approach to allow the codes to emerge from the data. This was an iterative process involving regular team discussions, enabling our ten initial CMOCs to evolve, and either be refined or refuted, and new CMOCs were developed to further uncover causal links between contexts, mechanisms and outcomes. Once a CMOC was established, all documents were re-read in detail for confirmation or disconfirmation. This process continued until the research team was in agreement that we had captured enough data to further develop and refine the programme theory. At this stage, we had a list of 22 CMOCs that were discussed in-depth during a face-to-face meeting between three of the authors (KI, AR, ER) and were categorised under four overarching themes, as presented in the results.

The second stage of synthesis involved engagement with key stakeholders to test and refine CMOCs. The rationale for involving relevant stakeholders was that they can help identify priorities, understand the problem and help find solutions that could make a difference to future implementation in the real world [65]. Realist interviews and focus groups with key stakeholders were used to ‘test’ the findings from the programme theory identified. Stakeholders provided feedback and discussion around the CMOCs during five focus group discussions and three individual interviews with a total of 26 health care professionals based at UK general practices (eight practice pharmacists, seven GPs, five advanced nurse practitioners, two frailty practitioners who were also social prescribers, two medical students on placement in a general practice, an advanced physiotherapy practitioner and a dietician). In addition, we conducted individual interviews with ten patients (aged 65 and over) and three informal carers to test the CMOCs. A further 11 new CMOCs were added to the list of 22 and underwent further iterations to the wording in order to ensure the context, mechanisms and outcomes were fully captured. When no further iterations were emerging, the research team agreed theoretical saturation had been reached and our programme theory was finalised.

The third stage involved going back to all included documents and notes from the stakeholder discussions and mapping them against the list of CMOCs for a final check. This helped us to see how relevant each CMOCs was to the dataset and check whether any important details had been missed. The final list of CMOCs alongside each data source is shown in Table 3. The next stage was to use the final programme theory to develop recommendations for primary care practice from an implementation perspective, drawing on Normalisation Process Theory (NPT) [66] (see Table 4). NPT focuses on what people do and how they work to address the issue of how interventions are adopted, embedded, and integrated into organisational routines. NPT explains this with reference to four constructs: coherence or sense making, cognitive participation, collective action, reflexive monitoring [66]. The mechanisms identified through our programme theory were mapped against the four constructs of NPT to aid clinicians and researchers to design services or interventions that could facilitate a successful implementation of MDT medication review and deprescribing process in primary care.

**Table 3:** Themes and Context Mechanism Outcome Configurations (CMOCs)

Theme and subthemes	Context Mechanism Outcome Configurations (CMOC)	Source
**Healthcare professional roles, responsibilities and relationships**	1. When conducting a multidisciplinary medication review (c), having a clear process with defined roles and responsibilities (m) can lead to higher acceptance rates of deprescribing recommendations by health care professionals leading to a safe reduction in number of medications taken by older people (o)	Campin et al. (2017) Stuhec et al. (2021) Romskaug et al. (2019) Van de Meer et al. (2019) Cardwell et al. (2020) (the opposite CMOC was confirmed) Clark et al. (2003)
	2. When a medication review is carried out in primary care (c), if the pharmacists are involved and well-integrated within the multidisciplinary team (m) there will be a reduction in inappropriate prescribing (o) because integration will aid decision-making through closer relationships and better communication between HCPs (o)	Lenaghan et al. (2007) Parkinson et al. (2021) Stuhec et al. (2021) Denneboom et al. (2007 (a) (b) Van de Meer (2019) Lenander et al. (2014) Khera e al (2019) Hazen et al. (2019) Clark et al. (2020) the opposite CMOC was confirmed) Trenaman et al. (2022) Jamieson et al. (2023) (the opposite CMOC was confirmed ) Stakeholder consultation
	3. When medication review involves a multidisciplinary approach (c), lack of trusted and healthy relationships between GPs and Pharmacists (m), leads to less deprescribing recommendations accepted and agreed resulting in unsuccessful deprescribing processes (o)	Bryant et al. (2010) Clark et al. (2020) Jamieson et al. (2023) Stakeholder consultation
	4. When a multidisciplinary medication review is carried out (c), if a pharmacist is involved in the process (m), then this gives health care professionals greater confidence to deprescribe medication (o)	Stakeholder consultation
	5. When conducting medication reviews (c), if GPs are actively involved and engaged in the process (m), then the pharmacists' recommendations are more likely to be accepted by GPs and communicated to patients (o)	Parkinson et al. (2021) Jamieson et al. (2023) (the opposite CMOC was confirmed)
	6. When pharmacists make deprescribing recommendations to the patients’ GP (c), there will be higher acceptance rates of the recommendations by GPs (o) if the recommendations are related to safety issues (i.e., potentially inappropriate or high-risk prescribing) rather than cost (m)	Cardwell et al. 2020
	7. When delivering medication reviews to older people (c), involving and utilising the skills of more healthcare professionals (e.g. HCAs, pharmacy technicians, social prescribers) (m) could make the medication review process more efficient and reach out to more patients (o)	Stakeholder consultation
	8. When medication reviews are conducted in collaboration between a health care professional from primary care and a geriatrician (c) combining the strengths of both specialties (m) there are more drug withdrawals and reduced dosages (o) resulting in positive effects on patients’ health-related quality of life (o)	Romskaug et al (2020)
	9. When conducting medication reviews within primary care, (for example, following patient discharge from hospital) (c), improving information continuity between primary and secondary care (m) and involving patients’ secondary care consultants (m) could support primary care teams to have more informed discussions with patients about any changes in their medications (o)	Stakeholder consultation Sheehan et al. (2022) Bayliss et al. (2022)
	10. Clear planning and recording of the rationale for prescribing, treatment goals, and length of treatment at the point of prescribing (m) could support HCPs in their conversations with patients about their medicines and support patients to anticipate, prepare for and engage in discussion around the continuing need for medications and facilitate their informed decision-making about deprescribing medicines (o) as part of the medication review process (c)	Stakeholder consultation
**Healthcare professional training and education**	11. When conducting a medication review by the MDT (c), providing training that incorporates experiential learning theory and collaborative direct patient care (m) leads to improvement in their knowledge and skills of polypharmacy and complex medication management, and significant reduction in the number of medications prescribed to older patients (o)	Mecca et al. (2019)
	12. When conducting a medication review by the MDT (c), providing Goal Attainment Scaling training focusing on individualized goal-setting and measurement among older patients (m), leads to a reduction in the number of medication prescribed (o)	Verdoorn et al. (2019)
**Format and process of the medication review: Efficiency of a multidisciplinary process**	13. When carrying out a medication review for older people (c), using an explicit or implicit tool to help identify any potentially unnecessary or inappropriate medications (m), may assist HCPs to make recommendations to deprescribe, resulting in a reduction in numbers of prescribed medications (o)	Denneboom et al. (2007) (a) (b) Williams et al. (2004) Campins et al. (2017) Mecca et al. (2022) Khera et al. (2019) Foubert et al. (2019)
	14. When medication reviews are conducted by GPs and pharmacists in collaboration (c), they are unlikely to carry out and sustain engagement in the process (o) if it is too time-consuming to deliver (m)	Leendertse et al. (2013) Sheehan et al. (2022) Bayliss et al. (2022)
	15. If a medication review involves patients with complex needs (c), then the co-location of pharmacists in primary care settings allows face-to-face communication (m) which facilitates timely shared-decision making between HCPs (o)	Dalin et al. (2019) (the opposite CMOC was confirmed) Van de Meer et al. (2018) (the opposite CMOC was confirmed) Cardwell et al. (2020) Clark et al. (2020) Stakeholder consultation
	16. When conducting medication review by the pharmacist (c), if a pharmacist has access to patient records (m), then they are more likely to be able to make, document and share better-informed deprescribing recommendations to other HCPs in a timely and more efficient way, leading to higher implementation of their deprescribing recommendations (o)	Oboh et al. (2018) Leendertse et al. (2013)( the opposite CMOC was confirmed) Bryant et al.(2010) Clark 2003 et al. (the opposite CMOC was confirmed) Jamieson et al. (the opposite CMOC was confirmed)
	17. When multidisciplinary teams are communicating to make decisions following a medication review (c), asynchronous communication using electronic tasking and messaging services is a useful and efficient means of communication	Stakeholder consultation
**Format and process of the medication review: Mode of communication with patients**	18. When HCPs conduct medication reviews (c), involving patients through face-to-face communication facilitates shared-decision making (m), leading to the reduction in medicines related-problems (o)	Oboh et al. (2018) Hazen et al. (2019) Trenaman et al. (2022) Stakeholder consultation
	19. When a pharmacist invites patients for a medication review (c), patients are unlikely to accept and engage in the process (o) if they have high treatment burden and have frequent visits to healthcare professionals, because they may have been reluctant to attend an additional appointment for the purpose of pharmacist review. Alignment of face-to-face medication review with other routine visits to reduce burden on patients could be useful (m)	Cardwell et al. (2020)
	20. When an HCP conducts a medication review (c) the mode of communication should be tailored to the individual needs of the patient and their families (m) in order to involve and engage them in the process, which is likely to lead to a reduction in prescribing (o)	Stakeholder consultation
**Format and process of the medication review: Patient follow-up support**	21. When conducting a medication review (c), if a scheduled follow-up with the patient is part of the process (m), there is more likely to be a reduction in number of inappropriate medications (o)	Hazen et al. (2019) Khera et al (2019) Lenaghan et al (2007) Trenaman et al et al (2022) Stakeholder consultation
	22. When conducting medication review (c) patients are more likely to have the confidence to accept and implement the deprescribing interventions (o) if deprescribing is offered as trial off medication that can be restarted anytime if needed (a ‘drug holiday’), with a planned follow-up with an HCP in place (m)	Stakeholder consultation
	23. After conducting medication review and agreeing with a patient on changes in medication regime (c), lack of engagement and poor follow-up by HCPs (m) could lead patients to feel a sense of abandonment (c) leading to not meeting patients' expectations and lack of continuity of care (o) affecting relationships with HCPs (o)	Bryant et al. (2010) Stakeholder consultation
**Involvement and education of patients and informal carers**	24. When pharmacists or advanced nurse practitioners invite patients for medication reviews (c), older people are less likely to participate and engage in the process and accept deprescribing recommendations (o) if they are unfamiliar with the role of pharmacists and advanced nurse practitioners (m)	Stakeholder consultation
	25. When conducting a medication review with a patient (c), if the patient has good understanding of the rationale of stopping a medication (m), they are more likely to accept, carry out and sustain HCPs recommendations (o)	Oboh et al (2018) Fixen et al. (2022) Stakeholder consultation
	26. When conducting medication reviews with high risk patients (c), educating patients and involving carers in reasons and decisions about medications mean they feel more engaged in the process (m) leading to accepting deprescribing recommendations and a reduction of the number of medications (o)	Lenaghan et al. (2007) Fixen et al. (2022) Stakeholder consultation
	27. When conducting a medication review (c), focusing on patient preferences and priorities (m), means patients are more engaged in in the process leading to acceptance in any changes in their medication regime (o)	Oboh et al (2018) Parkinson et al (2021) Hazen et al (2019) Verdoorn et al. (2019) Stakeholder consultation
	28. When communicating deprescribing decisions (c), patients are more likely to accept the recommendations (o) if they have a trusted relationship with the HCP (m)	Stuhec et al (2021) Stakeholder consultation
	29. When conducting medication reviews in primary care (c), patients are less likely to accept deprescribing recommendations from HCPs in primary care (o) if medication are prescribed by a consultant because patients believe they have specialist knowledge and therefore may be of the opinion that the medicines are to be taken indefinitely	Stakeholder consultation
	30. When having a medication review (c), if a patient feels anxious about the consequences of stopping a medication or views them as essential to their well-being (m) then they are less likely to accept deprescribing recommendations from health care professionals (o)	Stakeholder consultation
	31. When conducting a medication review (c) starting with a simple deprescribing change that can lead to noticeable improvements in symptoms by patients (‘quick win’) (m), can lead to patients being more likely to accept and implement further deprescribing recommendations (o)	Stakeholder consultation
	32. When conducting medication reviews with patients who have informal carers, including those living with cognitive impairment, (c), if informal carers are involved and engaged in the process (m), this will help the patient to feel more supported to implement any changes in medication (o)	Sheehen et al (2022) Stakeholder consultation
	33. When conducting medication review(c) informal carers are more likely to engage in a medication review and deprescribing process if they experience treatment burden related to medication management of their relatives (m), leading to increase in uptake of deprescribing recommendations (o)	Stakeholder consultation

**Table 4:** Recommendations for practice, based on Normalisation Process Theory (NPT) [66]

NPT construct	Definition of NPT construct	Recommendations
Coherence/sense-making	The extent to which study participants made sense of, and had a clear knowledge and understanding of the intervention	- Education for patients and carers on the rationale for deprescribing medications - HCPs training (experiential, MDT, direct patient care tasks) - Clear roles and responsibilities of the MDT team members - Familiarity of the role of pharmacists within primary care
Cognitive Participation	The extent to which participants bought into the intervention, engaged with it and committed to it	- Integration and co-location of pharmacists in primary care teams - Utilising the skills of different HCPs (e.g. nurses, social prescribers, frailty practitioners) - Involvement of informal carers - Building trusting relationship between the different HCPs
Collective Action	The allocation of organisational and personal resources to interventions, how the intervention was operationalised and the definition of roles and responsibilities	- Prioritising high-risk patients using practice systems - Offering deprescribing as a trial off medication or ‘drug holiday’ - Start with ‘quick wins’ - Tailored mode of communication (Face-to-face appointments vs telephone and home visits) - Taking into account patient preferences and goals (person-centred approach) - Aligning structured medication reviews with other appointments - Using deprescribing tools (eg STOPP/START) - Good communication between the team members (asynchronous vs synchronous) - Access to and documentation in medical records
Reflexive Monitoring	The extent to which the interventions were subjected to appraisal and evaluation, assessments of interventional impact, and processes of reflection, learning, and refinement to ensure sustained change	- Monitoring and follow up of patients

## Results

**Fig. 2:**
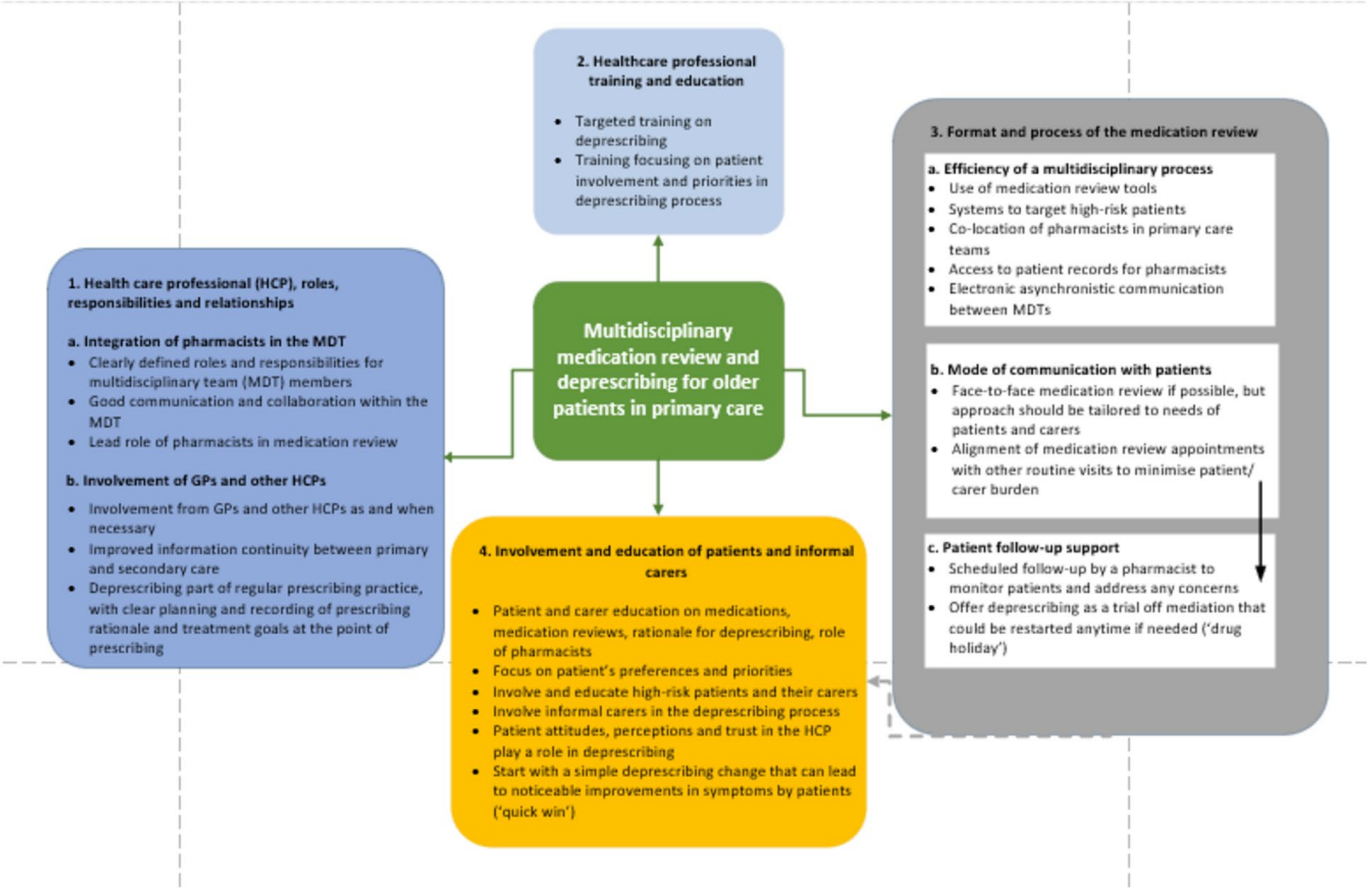
Final programme theory: Multidisciplinary medication review and deprescribing for older people in primary care

In total 28 documents were included. They were published between 2003–2023 and used a range of intervention designs, including ten Randomised Controlled Trials and four pilot studies. The majority of studies were conducted in the Netherlands, USA, Canada and New Zealand. We provide a narrative overview of the findings extracted in the 33 CMOCs (see Table 3) developed through our analysis, categorised under four themes (see Fig. 2). Our analysis identified potential intervention strategies and mechanisms that could facilitate a successful multidisciplinary approach for deprescribing in primary care for older people, as measured by the effective implementation of the approach and process, and patient and/or staff outcomes.

### Health care professional roles, responsibilities and relationships

Our review has identified the need for clearly defined roles and responsibilities for multidisciplinary team members and the importance of the communication and relationships within the team. In summary, when pharmacists are well-integrated into the primary care team and take the lead on conducting medication reviews, with the involvement of GPs and other health care professionals (HCPs) as appropriate, this facilitates better communication and decision-making within the team, leading to more successful implementation of deprescribing.

#### Integration of pharmacists in the MDT

A clear medication review and deprescribing process with defined roles and responsibilities of HCPs can lead to higher acceptance rates of deprescribing recommendations by GPs, with a reduction in the number of medications taken by older people (CMOC 1). This involved a pre-defined workflow plan for the roles of different HCPs at each stage of the process, mainly with pharmacists leading the medication review and GPs having the final decision about the recommendations. When pharmacists lead medication reviews, there is likely to be a reduction in inappropriate prescribing if they are well-integrated within the multidisciplinary team as this can facilitate closer relationships and better communication between HCPs, aiding decision-making (CMOC 2). Evidence was identified for the reverse, as lack of trusted and healthy relationships between GPs and pharmacists can lead to less pharmacist-initiated deprescribing recommendations being accepted and agreed by GPs (CMOC 3). Involvement of pharmacists in the medication review process is also reported to give health care professionals greater confidence to deprescribe unnecessary medications (CMOC 4). This was supported by the stakeholder consultation, which identified that experienced pharmacists who are well-integrated into the primary care team frequently take the lead on deprescribing decisions and consult with GPs when a patient has complex needs. Equally when a GP or advanced nurse practitioner takes the lead on a decision to deprescribe they consult with the practice pharmacist for advice when necessary.

#### Involvement of GPs and other HCPs

The importance of the involvement of GPs in the process was also identified through our review. When medication reviews are led by pharmacists, if GPs are actively involved and engaged in the discussions about patients’ medications, they are more likely to accept and discuss with patients any medication changes recommended by pharmacists (CMOC 5). In relation to communication between pharmacists and GPs where pharmacists are not integrated into the primary care team, GPs are more likely to accept pharmacist recommendations to deprescribe if they relate to issues with safety (i.e., potentially inappropriate or high-risk prescribing) rather than cost (CMOC 6). In addition to pharmacists and GPs playing a key role, our stakeholder consultation identified that involving and utilising the skills of other healthcare professionals (e.g., health care assistants, pharmacy technicians, social prescribers, frailty practitioners) could make the medication review process more efficient and reach out to more patients (CMOC 7). For example, health care assistants can complete measurements such as blood pressure, pharmacy technicians can provide advice on medicines to some patients with less complex needs, social prescribers can help to address alternatives to prescribing medication in relation to well-being and mental health, and frailty practitioners can conduct medication reviews as part of holistic needs assessments for those living with frailty.

Another approach to multidisciplinary medication reviews involved a collaboration between geriatricians and GPs. Suggestions from geriatricians for changes in medications following a comprehensive geriatric assessment of patients gives GPs confidence to implement medication changes. The close and trusting relationship that GPs have with the patient means they are in an ideal role to follow up patients to monitor medication changes. Findings showed that collaboration between a GP and a geriatrician can lead to more medicines being stopped and doses reduced resulting in positive effects on patients’ health-related quality of life (CMOC 8).

A key theme from our stakeholder consultation focused on the challenges of information continuity between primary and secondary care. When conducting medication reviews within primary care, for example following patient discharge from hospital, improving information continuity between primary and secondary care and involving patients’ secondary care consultants were suggested as ways to support primary care teams to have more informed discussions with patients about any changes in their medications (CMOC 9). Stakeholder consultation strongly suggested that deprescribing should be considered as part of regular prescribing practice in primary and secondary care. Clear planning and recording of the rationale for prescribing, treatment goals, and length of treatment at the point of prescribing could support HCPs in their conversations with patients about their medicines when medication reviews are due. This could also support patients to anticipate, prepare for and engage in discussion around the continuing need for medications and facilitate their informed decision-making about deprescribing medicines (CMOC 10).

### Healthcare professional training and education

Deprescribing is a complex process and many HCPs may experience issues with confidence in stopping medications safely, highlighting the need for training and education to support them in their deprescribing endeavours [67]. This was reflected in the findings from our stakeholder consultation as some HCPs expressed concern about the potential risks of deprescribing for patients, particularly older patients with frailty, hampered by a lack of clear clinical guidelines on deprescribing. Education on prescribing and deprescribing skills should run from “entry-to-practice” through the career of HCPs and should continue to be part of their professional development [68]. Our review identified two studies which reported that deprescribing decisions and improvements in patient and medication-related outcomes can be facilitated by training and education for HCPs that focus on patient involvement in deprescribing.

In the first study an interprofessional educational intervention incorporating experiential learning theory, team discussion and collaborative direct patient care, involving patients and carers in shared decision-making, led to improvements in the knowledge and skills of HCPs regarding polypharmacy and complex medication management, and resulted in a reduction in the number of medications, dose, and/or frequency of medications prescribed to older patients [41] (CMOC 11). The experiential learning model included (1) patient selection and completion of a medication review, (2) acquisition of new knowledge and concepts in a pre-clinic conference, (3) practical application in a shared medical appointment with patients and an individual appointment, and (4) synthesising information with an interprofessional team. Working as part of a team and being able to draw on the expertise and experience of colleagues helped some healthcare professionals overcome a lack of confidence in their own skills and experience. The second study reported that training for pharmacists based on the use of Goal Attainment Scaling (GAS) in clinical medication reviews, an individualised goal-setting and measurement approach aimed at patients with multiple health conditions, can lead to higher implementation rates of recommendations for drug-related problems compared to non-GAS related drug-related problem [49] (CMOC 12). Reduction in number of pills taken was one of the most prevalent goals identified by older people, attained by 20%.

### Format and process of the medication review

The format and process of multidisciplinary medication reviews and deprescribing are important elements in relation to outcomes. Specifically, this relates to factors that can facilitate the efficiency of a multidisciplinary approach to medication review and deprescribing, mode of communication with patients and planned follow-up with patients.

#### Efficiency of a multidisciplinary process

Our review has identified the importance of the resource efficiency of the medication review and deprescribing process for HCPs involved. In summary, the efficiency of a multidisciplinary medication review and deprescribing process can be facilitated by the use of medication review tools, systems to target high-risk patients, the co-location of pharmacists in primary care teams with access to patients’ medical records, and electronic asynchronistic communication.

In relation to time and resources, our review identifies several factors that can facilitate the efficiency of the process of medication review and deprescribing. Use of explicit or implicit tools to help identify any potentially unnecessary or inappropriate medications may assist HCPs to make recommendations to deprescribe, resulting in a reduction in numbers of prescribed medications (CMOC 13). (Refer to Table 2 for further details of the tools used).

Our review identified that GPs and pharmacists are less likely to carry out and sustain engagement in the process of a proactive multidisciplinary medication review if the process is too time-consuming for those delivering it (CMOC 14). Our stakeholder consultation highlighted the importance of practices having systems in place to carry out proactive as well as reactive medication reviews, by identifying high risk patients and prioritising those who need urgent medication reviews. However, there was no clear evidence from the review to suggest the best way in which patients should be targeted, for example based on level of frailty or types of medication. In the two studies targeting older people with frailty, one showed no difference [64] and the other did show a difference [45], with the main variation being the process of the medication review, and the integration and relationship between the MDT, indicating that collaborative working is particularly important for patients with complex needs. Importantly, the co-location of pharmacists in primary care settings allows face-to-face communication with GPs and other members of the MDT about high-risk patients or those with complex needs which facilitates timely shared-decision making (CMOC 15). Access to patient records, allows those conducting the medication review (e.g. pharmacists) to make, document and share better-informed deprescribing recommendations with other HCPs in a timely and more efficient way, leading to a higher implementation rate of their deprescribing recommendations (CMOC 16). Based on our stakeholder consultations, asynchronistic communication using electronic tasking and messaging services could also be a useful and efficient means of communication to make decisions as a multidisciplinary team (CMOC 17).

#### Mode of communication with patients

Whether medication reviews are conducted in-person, on the phone or virtually can impact on engagement of patients in the process and their decision to continue or stop medications. 

A shared decision-making approach involves clinicians and patients sharing the best available evidence when faced with the task of making decisions, and where patients are supported to consider options, to achieve informed preferences [69]. Studies included in this review highlight that involving patients through face-to-face communication during medication reviews facilitates shared-decision making, leading to a reduction in medicines related-problems (CMOC 18). Our review identified that patients with high treatment burden are less likely to attend and engage in primary care appointments if they have to attend an additional appointment for the purpose of medication review (CMOC 19). Treatment burden includes not only the workload of medication but all types of health care interventions, and its impact on patient functioning and well-being [70]. Alignment of face-to-face medication reviews with other routine visits is suggested as an approach that could reduce burden on patients who have a high treatment burden including frequent visits to healthcare professionals. Our stakeholder discussions also suggest the mode of communication during a medication review should be tailored to the individual needs of the patient and their informal carers in order to involve and engage them in the process (CMOC 20). For example, phone appointments are appropriate for some patients with limited mobility, and they impose less time burden for both patients and health care professionals. However, home visits, if feasible, are particularly useful for giving HCPs more context to older patients’ individual circumstances and medicines use, for example for patients living with frailty, carrying out medication review as part of a holistic approach by frailty practitioners during home visits may be an ideal approach to minimise burden, if primary care teams have the expertise and resources.

#### Patient follow-up support

Having a process in place for health care professionals to follow up and monitor patients, and provide support after initiating changes in medications, is key to facilitating and sustaining deprescribing, fostering continuity of care and trusted relationships between patients and HCPs. Our stakeholder consultation identified that pharmacists or advanced nurse practitioners are best placed to carry out a follow-up, as GPs have less time and capacity for this task.

Studies show that medication reviews including a scheduled follow-up by a pharmacist (either by phone or in person) to monitor and address any medication related issues are likely to lead to a reduction in the number of inappropriate medications prescribed to the patient (CMOC 21). In relation to this, patients are more likely to have the confidence to accept and stop a medication when pharmacists offer deprescribing as a trial off medication that could be restarted anytime if needed (‘drug holiday’), and a planned follow-up with an HCP is in place (CMOC 22). In the included studies, evidence was suggested for the reverse; lack of engagement and poor follow-up by HCPs after agreeing on changes in medication regime with patients, can lead to a negative impact on patients’ quality of life and relationships with HCPs. This was attributed to patients feeling a sense of abandonment due to unmet expectations and lack of continuity of care [37] (CMOC 23).

### Involvement and education of patients and informal carers

Involvement and education of patients and their relatives or informal carers in the process of a medication review and deprescribing is a key facilitating factor. Education about the indications of medicines, the purpose of regular medication reviews, the rationale for deprescribing and the role of pharmacists are areas that can facilitate the process of deprescribing.

When older patients are unfamiliar with the role of pharmacists and advanced nurse practitioners within primary care, they are less likely to participate and engage in a medication review when invited by a pharmacist or advanced nurse practitioners and are less likely to accept deprescribing recommendations (CMOC 24). When a patient has a good understanding of the rationale for stopping a medication, they are more likely to accept, carry out and sustain HCP's recommendations as part of a medication review (CMOC 25). In particular, when high-risk patients are involved in medication reviews, educating and involving both patients and informal carers on reasons and decisions about medications may mean they feel more engaged in the process leading to higher rates of acceptance of deprescribing recommendations and a reduction in the number of medications (CMOC 26). When a health care professional focuses on patient preferences and priorities during a medication review, this leads to patients feeling more engaged in the process meaning they are more likely to accept any changes in their medication regime (CMOC 27).

Patients are more likely to accept deprescribing recommendations when they come from a trusted HCP (CMOC 28). Our stakeholder consultation suggested that when conducting medication reviews in primary care, patients are hesitant to accept deprescribing recommendations from GPs or pharmacists if medications have been started by a secondary care consultant because patients trust that they have specialist knowledge and therefore may believe the medicines are to be taken indefinitely (CMOC 29). Transitions between primary and secondary care can result in gaps in documentation and communcation of changes made to patient treatment plans and can hinder HCPs understanding of patients’ current medication regimens and health needs, therefore obstructing the process of medicines optimisation and deprescribing. Attitudes and perceptions about deprescribing and patients’ relationship and trust of HCPs involved in the medication review process play an important role. Research suggests that patients are open-minded about deprescribing and are willing to stop medications if recommended by their doctors [71]. Stakeholder consultation suggested that if a patient feels anxious about the consequences of stopping a medication or views medications as crucial to their well-being then they are less likely to accept deprescribing recommendations from health care professionals (CMOC 30). This could be addressed by engaging patients in the deprescribing process through a ‘quick win’ approach (CMOC 31) whereby HCPs start the deprescribing process with a simple deprescribing change that can lead to either no change or noticeable improvements in symptoms by patients, and so are more likely to accept and implement further deprescribing recommendations when suggested by their HCPs in future medication review appointments.

Patients’ willingness to engage with and consider deprescribing may be shaped by how they perceive the value of their medications and the involvement of their families and carers [27]. Involving informal carers, including those caring for people living with cognitive impairment, in the medication review discussions (for example using resources such as educational brochures) can help patients to feel more supported to implement any changes in medications (CMOC 32). Based on our stakeholder consultation, relatives and informal carers of patients are more likely to engage in a medication review and deprescribing process if they experience treatment burden related to the medication management of their relatives, potentially leading to an increase in uptake of deprescribing recommendations (CMOC 33).

## Discussion

This realist review and synthesis has identified mechanisms, categorised under four overarching themes, that contribute to the success of multidisciplinary medication reviews and deprescribing interventions for older people within primary care. First, deprescribing interventions are more likely to work when collaboration exists between well-integrated multidisciplinary teams, with clear roles and responsibilities, and in particular where pharmacists take a lead role, but also utilising the strengths of other MDT members. Second, we identified that HCP training focusing on working as part of an MDT to support decisions about deprescribing as well as on involving patients and carers in shared-decision making about their medications could facilitate successful deprescribing interventions. Third, the format and process of the medication review is an important consideration in designing successful deprescribing interventions. Key aspects for considerations include appropriate systems of digital and face-to-face communication between the MDT members, co-location of team members and access to patient records, systems to target high risk patients and the use of tools to support medication reviews. The mode of communication with patients is also important with face-to-face appointments deemed ideal for discussing deprescribing, but with the ability to tailor this according to patient and carer needs. For example, home visits should be considered if possible, for patients living with frailty or without means to attend in-person, whereas telephone appointments could be appropriate for patients with less complex needs. Scheduled follow-up by a pharmacist (either by phone or in person) to monitor and address any medication related issues is also essential. Fourth, the involvement of patients and carers in the process of deprescribing is key, facilitated by trust in HCPs and continuity of care. Taking account of patient preferences and priorities, patient education including explaining the rationale for deprescribing, and involving carers, particularly where patients have cognitive impairment, can facilitate patient engagement and shared-decision making in the deprescribing process. Continuity of care is key and having systems for scheduled follow-up and monitoring should be in place.

Research indicates that detail on how deprescribing activities are delivered has previously been under-reported, making it challenging to apply evidence on deprescribing interventions in clinical practice [72]. Taking a novel approach, we draw on Normalisation Process Theory (NPT) [66] as a framework for our realist review, enabling the identification of a number of recommendations that could increase the success of implementing deprescribing in primary care (see Table 4). We discuss these below, with reference to the four NPT constructs: coherence or sense making, cognitive participation, collective action and reflexive monitoring.

### Coherence and sense making

#### Targeted training to increase HCP confidence

In relation to the NPT construct of coherence, referring to the extent to which study participants made sense of, and had a clear knowledge and understanding of the intervention [66], our review found that increasing confidence of HCPs in deprescribing can be achieved through targeted training and education programmes. Integrating shared-decision principles, goal setting, and experiential learning that includes practise in working as part of an MDT and applying knowledge in practice with patients can be beneficial. However, the availability of simulation-based training or other team training curricula, modules, and facilities focused on medicine management and deprescribing is rather haphazard [73]. A challenge for modern healthcare is making the provision and evidencing of systematic training in team skills a requirement of training and appraisal, rather than an option [74]. A qualitative synthesis of research investigating multidisciplinary primary care team working found that investing time and resources towards team building was beneficial [75]. This is increasingly important as the primary care workforce in the UK and elsewhere continues to diversify, including a greater number of different professions with prescribing rights. Deprescribing interventions designed to improve team working and communication are needed, with the potential to apply learning from the way MDTs work in other specialities such as surgery and oncology, within the primary care working environment.

####  Implementing a supportive infrastructure with clear MDT roles and responsibilities

Another important mechanism identified by our review, to support coherence of deprescribing interventions is related to the roles and responsibilities of the MDT members. It has been argued that a multidisciplinary approach can result in diffusion of responsibility, a reluctance to stop medicines prescribed by others and a lack of confidence to intervene in complex medication regimens [2]. However, as our review has identified, a supportive system that provides clear guidance around professional roles and responsibilities and that enables multidisciplinary working and communication, and continuity of care can address this [27]. Clarifying roles and responsibilities in the medication review and deprescribing process, and allocating sufficient resources to these roles, would provide the infrastructure to support and formalise the process, which may give healthcare professionals the “permission” to undertake it [27]. However, we acknowledge that deep-rooted hierarchical cultures within healthcare systems are an important factor here, that are unlikely to be an easy challenge to overcome. There is a need for a deprescribing competency framework to help define responsibilities against capabilities and skills. Work on this has already begun, for example a curricular framework for an interprofessional approach to deprescribing [76], standards of practice for polypharmacy and chronic disease medication reviews in general practice [77] have been developed, and a deprescribing competency framework in nursing for older people is also under development [78]. Using competency frameworks to allocate roles within the MDT team regarding deprescribing decisions could be beneficial however more research is needed in this area.

#### Patient and carer information and education

Informing and educating patients and their carers about the role of pharmacists, the purpose of medication reviews and the rationale for deprescribing, are key facilitators for patient and carer engagement and shared-decision making. A recent review of deprescribing interventions in primary care identified that most patient education focused on the patient’s medical condition, harm from medications and on a lack of evidence for medicine continuation in old age [79]. However, most of the available resources did not contain balanced information on both potential benefits and risks of deprescribing, and the majority required high health literacy levels making them inaccessible to many [80]. Co-designing materials with patients and carers is important to incorporate their personalised needs, given the mounting evidence that patient-centred care and shared-decision making can improve patient satisfaction, adherence, quality of life and overall health outcomes [81].

### Cognitive participation

#### Integrated multidisciplinary teams

In relation to the NPT construct of cognitive participation, referring to the extent to which participants bought into the intervention, engaged with it and committed to it [66], we found that the process of deprescribing can be facilitated by drawing on the strengths of different HCPs. This includes frailty practitioners, advanced nurse practitioners and social prescribers, as well as pharmacists. Pharmacists have a key role to play in deprescribing and our realist review has shown that integration and co-location of pharmacists within the primary care team is key to drive success of deprescribing. In the UK, pharmacists and other non-medical prescribers are increasingly working as part of a multidisciplinary primary care team to consult with and treat patients directly and improve outcomes related to medicines [82] and more countries are moving towards integrating pharmacists in primary care.

#### Maintaining continuity of care and information

Trust in HCPs and continuity of care are essential elements of medication review and deprescribing [27]. Our stakeholder consultation has indicated that positive relationships between HCPs and patients can facilitate deprescribing conversations and help HCPs to effectively implement deprescribing. The feeling of confidence or reassurance that the healthcare professional has the patients’ best interests at heart and that their decisions are grounded in an understanding of the patient, is a core component of effective healthcare [27]. To build trust between patients and HCPs it is essential to reach a shared understanding of the risks and benefits of medicines with tailored explanations through a consistent management plan that includes a planned follow-up to review any changes [27]. All forms of continuity (relational, informational and management) are important for building trust and successful engagement in deprescribing [83]. A patient-centred approach through actively identifying patient needs, enabling patient and carer involvement in deprescribing discussions and agreement on action plans are important mechanisms [27].

#### Considering deprescribing as a continuum

Deprescribing should be considered part of routine prescribing practice [84], and approached as a continuum beginning when a prescription is first initiated. It is important that goals of treatment are recorded at the point of prescribing to support patients and their health care professionals in future consultations to discuss the continuing need for medications [2]. Our realist stakeholder consultations highlighted that this could support health care professionals in their dialogue with patients about their medicines. It could also help patients to make informed decisions at the start of treatment and to weigh up the benefits and risks when considering tapering or stopping a medication. Prescribing, especially among older people with comorbidities, should incorporate the principles and practices of effective safety-netting into the patient management plan [85], and our review suggests that incorporating these safety-netting principles and practices into the medication review and deprescribing conversation is essential to protect both the patient and HCP. Collaborative decision-making with patients could mean sharing responsibility of deprescribing decisions between HCPs and patients, thus addressing some of the challenges HCPs face in terms of lack of guidelines and uncertainty about consequences of stopping medicines [27].

### Collective actions

#### Tailored mode of communication, documentation and delivery

Our review has identified a number of mechanisms in relation to the NPT construct of collective actions, referring to the allocation of organisational and personal resources, how the intervention was operationalised and the definition of roles and responsibilities [66]. These included open and good communication channels between MDT members (synchronous and asynchronous, including electronic tasking systems), tailored mode of delivery of medication review appropriate to patients’ needs, systems to identify and target high risk populations, accessing clinical records for accurate acquisition of patient information and better documentation of deprescribing plans and the use of tools to support medication reviews. Recent reviews have identified the range of implicit and explicit tools available for different stages in the process of medication review and deprescribing and further research is needed to assess their implementation in clinical practice [14, 86].

#### Considering patient and carer priorities and implementing strategies to develop trust in deprescribing decisions

There is evidence that older patients may be willing to stop a medication if recommended by their doctors [87], however our stakeholder consultation indicated that some HCPs might be hesitant to deprescribe medications initiated by hospital specialists and patients might feel some reliance on medications. It is therefore, important to identify patients’ attitudes towards deprescribing and define their goals of care by involving them in the medication decision-making and management process before suggesting reducing or stopping a medication. Our stakeholder analysis has identified that patient engagement and trust can be developed by offering deprescribing as a trial off medication, (‘drug holiday’) that can be monitored and restarted anytime if needed. Trust can also be developed by starting with simple deprescribing changes tailored to the individual patient, taking into consideration their priorities, that could lead to noticeable improvements in symptoms by patients (‘quick wins’). These gradual, incremental changes over time in partnership with the patient have been referred to as ‘tinkering’ [88]. Our findings support an ethnographic study that identified medication review as a complex and ongoing, collaborative process characterised by small, incremental changes, best in the context of relational continuity, as opposed to a ‘one-off’ activity [89] carried out by a single health care professional working in isolation. It is important this is taken into account when decisions are made about how care pathways and service delivery operate.

### Reflexive monitoring

#### Monitoring and follow up of patients

With reference to the NPT construct of reflexive monitoring, referring to the extent to which interventions are subject to evaluation, assessments of interventional impact, and processes of reflection, learning, and refinement to ensure sustained change [66], our review has found that having systems in place to monitor and follow-up patients following medication changes is essential. Robust follow-up plans should be agreed with patients, allowing continuity of care and support for patients who are concerned about negative consequences and withdrawal of care. Monitoring of symptoms/side-effects should be tailored to patients’ cases and may involve follow-up telephone or in-person appointments with a focus on the provision of support in relation to their priorities as well as the tracking of any withdrawal symptoms or physiological responses to deprescribing (e.g., blood pressure and cholesterol checks) [72]. Our review suggests that pharmacists, pharmacy technicians and nurse prescribers are best placed to monitor and follow-up patients, providing co-ordination and continuity of care. Management continuity can help reassure patients and healthcare professionals that any unwanted effects of medication changes will be managed [27].

### Strengths and limitations

The strength of our study lies in the realist review methods, ideal in revealing the underlying mechanisms that explain how an intervention works, or does not, for whom in relation to older patients, informal carers and health care professionals, under what circumstances and why [32], with valuable guidance from our multidisciplinary RMG and stakeholder consultation. Taking a novel approach, we used NPT as a framework for our realist review, to suggest mechanisms to facilitate the implementation of the deprescribing process in primary care. NPT has been found to provide a consistent representation and explanation of the processes of intervention implementation in primary care, irrespective of the focus of the intervention [90]. Our realist review focused on interventions targeting MDTs in primary care, and the majority did not generally include data from patients and carers. However, we have incorporated qualitative stakeholder consultation data from patients, carers and HCPs which has provided important detail to strengthen and extend our CMOCs. Although a few limitations should be considered when interpreting our findings. The UK context of our stakeholder consultations where pharmacists are becoming established members of the primary care team, with a prescribing role and increasingly responsible for conducting medication reviews, differs from the context of the majority of studies included in the review where pharmacists generally did not have a prescribing role, nor were they an established part of the primary care team, on the whole. Therefore, these different contexts must be taken into account when interpreting and applying the findings in clinical practice. Very few studies measured time-savings, which our review has shown is an important aspect for HCPs when conducting medication reviews and is particularly important given the increasing pressure primary care is currently experiencing in the UK and elsewhere.

## Conclusion

Our work has highlighted the complexity of medication review and deprescribing interventions. We have developed a programme theory and made recommendations that could support general practices to prioritise and implement deprescribing more efficiently. A multidisciplinary approach with pharmacists taking a lead role but in the context of a well-integrated and co-located team, with clear roles, good communication and collaboration, drawing on the strengths and expertise of other MDT members, and the involvement and engagement of patients and carers in the process are all key. It is important to have a clear process that identifies and targets high-risk populations (for example, those taking 10 or more medicines, referred to as hyper-polypharmacy, or on specific high-risk medications) for medication review, using appropriate modes for involving patients and their carers/family members in discussions about their medications, starting with quick wins and offering deprescribing as a drug holiday, and ensuring appropriate and tailored follow-up plans that allow continuity of care and management. Deprescribing is challenging due to lack of policies and guidelines as well as the complexity of the health care system and the complexity of patients, particularly those who are older and living with frailty. Therefore, it is crucial to use ‘out-of-the-box thinking’ and embrace innovative, non-traditional approaches that bypass systems-level barriers [91]. We hope that our programme theory and implementation strategies will contribute to an understanding of what good deprescribing practice looks like and inform the design of future interventions and services that successfully embed deprescribing in primary care.

### Supplementary Information

**Additional file 1: Supplementary file 1.** MODIFY realist review search strategy, 18^th^ March 2022.

**Additional file 2: Supplementary file 2.** List of items required when reporting a realist synthesis (RAMESES checklist).

**Additional file 3: Supplementary file 3.** Initial Programme Theory.

## Data Availability

The datasets used and/or analysed during the current study are available from the corresponding author on reasonable request.
