# Supplementary material for: What makes a multidisciplinary medication review and deprescribing intervention for older people work well in primary care? A realist review and synthesis
Source: BMC Geriatr. 2023 Sep 25;23:591. doi: 10.1186/s12877-023-04256-8 (PMC10519081; doi:10.1186/s12877-023-04256-8)
Supplement: Supplementary file 1 — Additional file 1: Supplementary file 1. MODIFY realist review search strategy, 18th March 2022. [file 12877_2023_4256_MOESM1_ESM.docx]

**Supplementary file 1:**

**MODIFY realist review search strategy, 18^th^ March 2022**

1. medication therapy management/

2. ((medici* or drug*) adj3 (management or reconcil* or optimi* or review)).mp. [mp=title, abstract, heading word, drug trade name, original title, device manufacturer, drug manufacturer, device trade name, keyword heading word, floating subheading word, candidate term word]

3. 1 or 2

4. aged/ or frail elderly/ or very elderly/

5. (aged or elder* or old* or geriatric*).mp. [mp=title, abstract, heading word, drug trade name, original title, device manufacturer, drug manufacturer, device trade name, keyword heading word, floating subheading word, candidate term word]

6. 4 or 5

7. 3 and 6

8. multidisciplinary team/ or collaborative care team/

9. (multidisciplinary or multi-disciplinary).mp. [mp=title, abstract, heading word, drug trade name, original title, device manufacturer, drug manufacturer, device trade name, keyword heading word, floating subheading word, candidate term word]

10. community pharmacist/ or pharmacist/

11. general practitioner/

12. nurse/ or exp advanced practice nurse/ or nurse consultant/

13. nurse*.mp. [mp=title, abstract, heading word, drug trade name, original title, device manufacturer, drug manufacturer, device trade name, keyword heading word, floating subheading word, candidate term word]

14. (general practitioner* or family doctor* or family physician*).mp. [mp=title, abstract, heading word, drug trade name, original title, device manufacturer, drug manufacturer, device trade name, keyword heading word, floating subheading word, candidate term word]

15. pharmacist*.mp. [mp=title, abstract, heading word, drug trade name, original title, device manufacturer, drug manufacturer, device trade name, keyword heading word, floating subheading word, candidate term word]

16. 8 or 9 or 10 or 11 or 12 or 13 or 14 or 15

17. 7 and 16

18. deprescription/

19. (deprescrib* or deprescription*).mp. [mp=title, abstract, heading word, drug trade name, original title, device manufacturer, drug manufacturer, device trade name, keyword heading word, floating subheading word, candidate term word]

20. ((medic* or drug*) adj3 (withdraw* or stop or discontinue or cessation)).mp. [mp=title, abstract, heading word, drug trade name, original title, device manufacturer, drug manufacturer, device trade name, keyword heading word, floating subheading word, candidate term word]

21. polypharmacy.mp. [mp=title, abstract, heading word, drug trade name, original title, device manufacturer, drug manufacturer, device trade name, keyword heading word, floating subheading word, candidate term word]

22. 18 or 19 or 20 or 21

23. 17 and 22

24. primary medical care/ or primary health care/

25. ((primary or family or general) adj3 (practi* or physician* or doctor*)).mp. [mp=title, abstract, heading word, drug trade name, original title, device manufacturer, drug manufacturer, device trade name, keyword heading word, floating subheading word, candidate term word]

26. (primary adj3 care).mp. [mp=title, abstract, heading word, drug trade name, original title, device manufacturer, drug manufacturer, device trade name, keyword heading word, floating subheading word, candidate term word]

27. 24 or 25 or 26

28. 23 and 27
