# Supplementary material for: What makes a multidisciplinary medication review and deprescribing intervention for older people work well in primary care? A realist review and synthesis
Source: BMC Geriatr. 2023 Sep 25;23:591. doi: 10.1186/s12877-023-04256-8 (PMC10519081; doi:10.1186/s12877-023-04256-8)
Supplement: Supplementary file 3 — Additional file 3: Supplementary file 3. Initial Programme Theory. [file 12877_2023_4256_MOESM3_ESM.docx]

**Supplementary file 3: Initial Programme Theory**

1. **Context-Mechanism-Outcome Configurations on the role of the health care professional and the multidisciplinary team**

If a primary health care professional (eg. GPs, ANPs, Practice pharmacists) conducts a medication review in collaboration with a multidisciplinary team, then they will feel more confident to take a decision to deprescribe because the responsibility will be shared as a team. This will lead to a safe reduction in unnecessary medications for older patients, resulting in better health outcomes.

If there is good communication between multidisciplinary health care professionals (eg. GPs, ANPs, Practice pharmacists) in the process of conducting a medication review, then health care professionals will feel more supported and more confident to deprescribe unnecessary medication because the responsibility will be shared as a team. This will lead to a safe reduction in unnecessary medications for older patients, resulting in better health outcomes.

If practice pharmacists are involved in or lead medication reviews in primary care, then the intervention will be delivered to more older adults because this addresses the time constraints and workload challenges of GPs and this has an effect on successful deprescribing interventions.

If primary care staff receive targeted training on deprescribing, then this will lead to higher rates of medication reviews and deprescribing because training could increase their skills, knowledge, confidence in managing and stopping inappropriate drugs. This will lead to a safe reduction in unnecessary medications for older patients, resulting in better health outcomes.

If medication reviews focus on specific classes of drugs, then it would be more feasible for primary care teams to identify and stop inappropriate drugs and closely monitor the impact of stopping them

1. **Context-Mechanism-Outcome Configurations on relationships and communication between health care professionals, patients, carers/ relatives**

If primary care professionals involve and educate patients and carers/ relatives about the reasons for deprescribing during a medication review, then patients will have a better understanding of the reasons for deprescribing and feel more engaged in the deprescribing process. Therefore, they will be more likely to continue with any changes in medication leading to better self-efficacy/ better health outcomes.

If primary care professionals involve patients in medication reviews and make shared decisions with the patients and carers/ relatives about deprescribing then patients will feel more engaged in the deprescribing process and be more likely to continue with any changes in medication leading to better self-efficacy/ better health outcomes.

If medication reviews focus on patient preferences and priorities, then patients would be engaged in the process of a multi-disciplinary medication review and deprescribing. This will mean they are more likely to sustain any changes in their medication regime resulting in better self-efficacy/better health outcomes.

If there is good communication between health care professionals, patients and carers/ relatives, then patients will feel more engaged in the process of a medication review and deprescribing and be more likely to sustain any changes in their medication regime resulting in better self-efficacy/ better health outcomes.

If primary care professionals regularly follow-up patients after deprescribing during a medication review, then patients will feel more supported in the deprescribing process and be more likely to continue with any changes in medication, leading to better self-efficacy/ better health outcomes.
